# *MEFV* gene mutations in neuro‐Behçet's disease and neuro‐Sweet disease

**DOI:** 10.1002/acn3.50937

**Published:** 2019-11-04

**Authors:** Hidehiro Ishikawa, Akihiro Shindo, Yuichiro Ii, Dai Kishida, Atsushi Niwa, Yamato Nishiguchi, Keita Matsuura, Natsuko Kato, Akane Mizutani, Kei Tachibana, Yoshinori Hirata, Hirofumi Matsuyama, Ai Ogawa‐Ito, Akira Taniguchi, Hidekazu Tomimoto

**Affiliations:** ^1^ Department of Neurology Mie University Graduate School of Medicine Mie Japan; ^2^ Department of Medicine (Neurology and Rheumatology) Shinshu University School of Medicine Nagano Japan

## Abstract

*Mediterranean fever (MEFV)* gene mutations are associated with familial Mediterranean fever (FMF). Recent studies have suggested that *MEFV* gene mutations may act as disease modifiers in neuro‐Behçet's (NBD) disease and neuro‐Sweet disease (NSD). We investigated *MEFV* genes and clinical features in 17 patients with NBD or NSD. *MEFV* gene mutations were frequently observed (70.6%). Headaches and exertional leg pain were associated with *MEFV* gene mutations (*P* < 0.05). Moreover, higher frequency of white matter lesions without sites predilection (*P* < 0.05) and non‐parenchymal lesions (*P* < 0.05) were also observed. *MEFV* gene mutations may be associated with particular findings and lesion sites.

## Introduction

Neuro‐Behçet's disease (NBD) and neuro‐Sweet disease (NSD) are neurological manifestations of Behçet's disease (BD) and Sweet disease, respectively, with occasional serious sequelae.^1^, ^2^ Considering the overlap in their symptoms, NSD and NBD can be regarded as forms of neuro‐neutrophilic disease (NND), a broad‐spectrum disorder caused by the hyperactivity of neutrophilic cells.^3^

Genetically, class I human leukocyte antigen (HLA) serotypes have been associated with BD (e.g., HLA‐B51)^4^ and NSD (e.g., HLA‐B54 and Cw1).^2^ According to recent reports, mutations in the Mediterranean fever (*MEFV)* gene, which is associated with familial Mediterranean fever (FMF),^5^ may act as disease modifiers for NBD^6^, ^7^ and NSD.^8^, ^9^ FMF, an autoinflammatory disease, is characterized by recurrent episodes of fever, serositis, arthritis, and dermal manifestations.^5^ BD and FMF share common symptoms, laboratory findings, and treatments.^10^ Colchicine is the mainstay of FMF treatment and is also one of the valid therapeutic options for BD. Therefore, there may be a pathophysiological link among NBD, NSD, and FMF. Clarifying the role of *MEFV* gene in NBD and NSD is important for elucidating the mechanism of central nervous system (CNS) inflammation and developing treatments.

We hypothesized that HLA class I subtypes and *MEFV* gene mutations might serve as susceptibility, or disease‐modifying factors in patients with CNS inflammation, resulting from NND. Furthermore, we hypothesized that specific clinical manifestations related to FMF^5^ could indicate *MEFV* gene mutations in patients with NBD or NSD.

Herein, we investigated FMF‐related symptoms and their associations with HLA class I alleles and *MEFV* gene mutations in patients with NBD or NSD.

## Materials and Methods

### Subjects

This retrospective study included 17 patients who visited Mie University Hospital between April 2014 and December 2018 and received confirmed NBD or NSD diagnosis. The International Consensus Recommendation criteria were used for NBD diagnosis,^1^ with a diagnosis of definite NBD when all of the following three criteria were met: (1) International Study Group (ISG) criteria for BD^11^; (2) neurological syndrome due BD and supported by relevant and characteristic abnormalities seen on neuroimaging or cerebrospinal fluid analysis; and (3) no better explanation for the neurological findings; and probable NBD when one of the following two criteria were met in the absence of a better explanation for the neurological findings: (1) neurological syndrome as in definite NBD, with systemic BD features but not satisfying the ISG criteria; (2) a non‐characteristic neurological syndrome occurring in the context of ISG criteria‐supported BD. NSD was diagnosed using the criteria proposed by Hisanaga et al in 2005^2^ and was designated as probable NSD when all of the following three criteria were met: (1) neurological features – highly systemic glucocorticoid responsive or sometimes spontaneously remitting, but frequently recurrent encephalitis or meningitis, usually accompanied by fever over 38°C; (2) dermatological features – painful or tender, dull red erythematous plaques or nodules preferentially occurring on the face, neck, upper limbs, and upper part of the trunk; (3) other features – absence of cutaneous vasculitis and thrombosis, which are seen in BD; and possible NSD with any neurological manifestations, either dermatologic features or HLA association (HLA‐Cw1 or B54 positive, B51 negative), and at least one item of ‘other features' described above. This study followed the Clinical Study Guidelines of the Ethics Committee of Mie University Hospital and was approved by its internal review board (approval number, 1756).

### MEFV gene analysis

A *MEFV* gene analysis was conducted in all patients. Mutations in the five hotspot regions (i.e., exons 1, 2, 3, 5, and 10) were assessed through polymerase chain reaction 12. Exon 2 was amplified in two overlapping PCR fragments, designated as exon 2a and exon 2b. Amplified PCR products were analyzed by direct sequencing (DNA Analyzer 3730xl; Applied Biosystems, Foster City, CA, USA). Primers for the PCR and sequence analysis were as follows: Exon 1F: 5′‐TCC TAC CAG AAG CCA GAC AG‐3′; Exon 1R: 5′‐TTC CTG AAC TAA AGT CAT CT‐3′; Exon 2aF: 5′‐GCA TCT GGT TGT CCT TCC AGA ATA TTC C‐3′; Exon 2aR: 5′‐CTT TCC CGA GGG CAG GTA CA‐3′; Exon 2bF: 5′‐CAG GCC GAG GTC CGG CTG CG‐3′; Exon 2bR: 5′‐CTT TCT CTG CAG CCG ATA TAA AGT AGG‐3′; Exon 3F: 5′‐GAA CTC GCA CAT CTC AGG C‐3′; Exon 3R: 5′‐AAG GCC CAG TGT GTC CAA GTG C‐3′; Exon 5F: 5′‐TAT CGC CTC CTG CTC TGG AAT C‐3′; Exon 5R: 5′‐CAC TGT GGG TCA CCA AGA CCA AG‐3′; Exon 10F: 5′‐CCG CAA AGA TTT GAC AGC TG‐3′; Exon 10R: 5′‐TGT TGG GCA TTC AGT CAG GC‐3′. Annealing temperatures were as follows: Exon 1: 52°C; Exon 2: 60°C; Exon 3: 60°C; Exon 5: 60°C; Exon 10: 60°C.

### Symptoms and laboratory data

In addition to neurological syndromes (i.e., headache, cognitive impairment, seizure, gait disturbance, and vascular manifestations), symptoms indicative of FMF (i.e., fever, peritonitis, pleuritis or pericarditis, monoarthritis, and exertional leg pain)^5^ or NND (i.e., ocular lesions, skin lesions, oral aphthae, benign genital ulcers, and pathergy reaction)^1^, ^2^ were assessed. The data of HLA genotypes and laboratory data on blood inflammatory markers, including white blood cell counts, C‐reactive protein levels, serum amyloid A levels, and erythrocyte sedimentation rate, were assessed. Inflammatory markers in the cerebrospinal fluid, such as high cellularity, protein, IgG index, or interleukin‐6 levels^1^ were also assessed.

### Neuroimaging data

**Figure 1:**
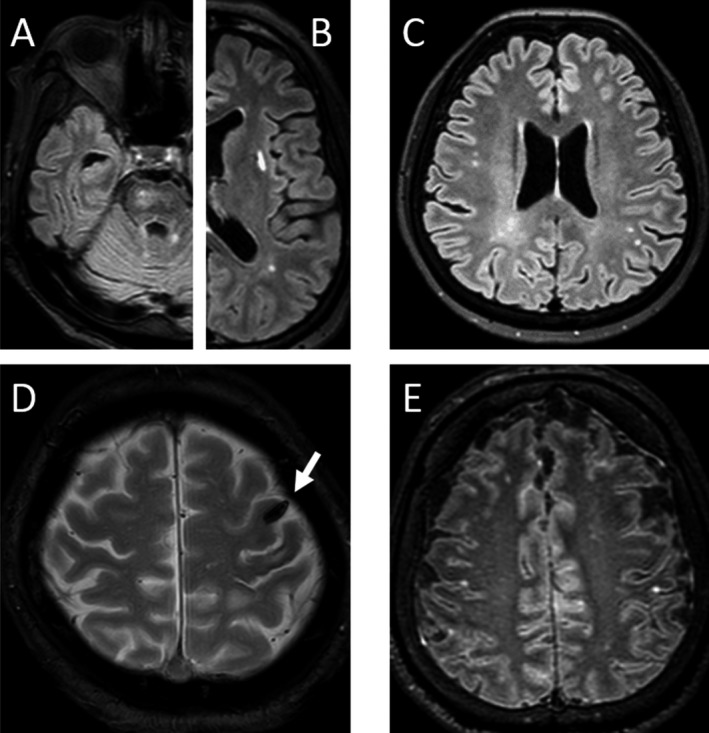
Representative magnetic resonance imaging (MRI) findings of lesion locations such as brain stem (A), basal ganglia (B), white matter without site predilection (C), and non‐parenchymal lesions (D, E). Brain stem, basal ganglia, and white matter lesions were assessed with fluid‐attenuated inversion recovery (FLAIR) images (A–C). Cerebral vein thrombosis was detected as hypo‐intensity on T2 star‐weighted image (D; arrow). Post‐contrast three‐dimensional FLAIR image revealed leptomeningeal enhancement compatible with acute meningeal syndrome (E).

Brain magnetic resonance imaging (MRI) was performed with a 3T MR scanner (Ingenia, Philips Health Care, Best, The Netherlands). All patients underwent 3D fluid‐attenuated inversion recovery (FLAIR), 3D T1‐weighted imaging (T1WI), T2‐weighted imaging, T2 star‐weighted imaging or susceptibility‐weighted imaging, diffusion‐weighted imaging, and MR angiography diagnostic imaging. Post‐gadolinium (Gd) FLAIR (FLAIR‐Gd) and Gd‐T1WI (T1WI‐Gd) were performed on 11 patients without renal failure. These patients provided written informed consent for the use of Gd. Regional brain lesion locations were categorized into brain stem (Fig. 1A), basal ganglia (Fig. 1B), white matter without sites predilection (Fig. 1C), non‐parenchymal lesions which included cerebral venous thrombosis (Fig. 1D) or acute meningeal syndrome (Fig. 1E)^1^, ^2^, ^3^, or lobar locations.

### Data analysis

Patients were divided into a *MEFV* mutation positive group (*MEFV*+) and negative group (*MEFV−*) and compared the frequency of HLA class I genotypes between the groups. Group‐wise differences in symptoms, laboratory data, and neuroimaging data were also assessed. All data analyses were performed using SPSS version 25 for Windows (IBM Corp.). To assess statistical differences between categorical variables, we used the chi‐square test. The significance level was set at 0.05 for all analyses.

## Results

The demographic, genetic, radiographic, and therapeutic data of patients are summarized in Table 1. Among the patients who underwent *MEFV* gene analysis, 12 (70.6%) displayed heterozygous or homozygous mutations. The frequencies of occurrence of HLA‐B51, HLA‐B54, and HLA‐Cw1 were 23.5%, 29.4%, and 52.9%, respectively. The most frequent symptoms were headache (76.5%) and oral mucosal lesions (76.5%), followed by incomplete joint disorders (64.7%). MRI revealed that the lesions in the brain stem (BS) (64.7%) were most common, followed by the white matter lesions, without predilection for a particular site (58.8%).

**Table 1:** Summary of genetic, radiographic, and therapeutic data

Case	Age/Sex	Diagnosis	*MEFV* mutations	HLA genotypes			MR lesions	Treatment	Response
				A	B	C			
1	34 M	Probable NBD	None	0206 3303	4002 4403	0304 1403	BS, BG	None	–
2	42 F	Possible NSD	None	0207 1101	4601 5201	0102 1202	Frontal lobe	SP	E
3	57 M	Probable NBD	None	2402 3201	4402 5101	0501 1402	BS, BG	None	–
4	60 M	Definite NBD	None	0201 2402	1501 5101	0304 1402	BS, WM	SP	E
								OS	NE
5	67 M	Possible NSD	None	0201 2402	3501 5901	0102 0303	Parietal lobe	SP	E
							Occipital lobe		
6	37 F	Possible NSD	E148Q	2402 2446	4002 5502	0102 0304	non‐parenchymal	SP	E
7	42 F	Probable NBD	E148Q	3101	4001 5101	0304 1402	BS, BG	none	–
8	46 F	Probable NBD	E148Q	2402	5101 5201	1202 1402	BS	SP	E
9	46 M	Probable NSD	E148Q, R202Q	0206 2402	4001 5401	0102 0702	BS, WM, non‐parenchymal	SP	E
								OS	E
10	48 F	Probable NBD	G304R	0206 2602	1501 5201	0303 1202	BS, WM	OS	E
11	51 F	Probable NSD	E84K	0201 0206	3902 5401	0102 0702	BS, WM	OS	E
								CL	E
12	61 M	Possible NSD	R202Q	2402 3303	4403 5401	0102 1403	BS, WM	SP	E
								OS	E
13	65 M	Possible NSD	E148Q	2402 2402	0702 5401	0102 0702	BS, WM	SP	E
14	53 F	Probable NBD	E148Q, P369S, R408Q	0201	1518	0704	WM, non‐parenchymal	CL	E
15	73 M	Probable NBD	E148Q	1101 3303	4403	1403	BS, WM, non‐parenchymal	none	‐
16	66 M	Possible NSD	L110P, E148Q (homo)	1101 2601	4002 5502	0102 0304	WM, non‐parenchymal	OS	E
17	71 M	Possible NSD	E148Q, P369S, R408Q	2402 2601	4002 5401	0102 0304	BG, WM, non‐parenchymal	OS	E

M, male; F, female; *MEFV*, Mediterranean fever gene; HLA, human leukocyte antigen; MR, magnetic resonance; N/A, not available, BS, brain stem; BG, basal ganglia; WM, white matter; S *P* = steroid pulse; OS, oral steroids; CL, colchicine; E, effective; NE, not effective.

A comparison of the clinical symptoms of the *MEFV positive (+)* and *MEFV negative *(−) groups is presented in Table 2. HLA class I serotypes were not significantly different between the *MEFV+* and *MEFV*− groups. Although insignificant, *MEFV*+ patients had B54 alleles frequently (41.7% vs. 0%, *P* = 0.086) and A02 alleles less frequently (33.3% vs. 80%, *P* = 0.079). Common symptoms in the *MEFV*+ group included headaches (100% vs. 40%, *P* = 0.003) and exertional leg pain (66.7% vs. 0%; *P* = 0.012). MRI findings indicated that *MEFV*+ patients exhibited a higher frequency of white matter lesions without site predilection (75% vs. 20%, *P* = 0.036) and non‐parenchymal lesions (50% vs. 0%, *P* = 0.049). There were no significant differences in laboratory data including inflammatory markers in blood and cerebrospinal fluid of patients classified as *MEFV*+ and *MEFV*−. Responses to the treatment were not significant between *MEFV*+ and *MEFV*− patients.

**Table 2:** Comparison of patients with or without *MEFV* gene mutations.

No. (%)			Total (*n* = 17)	MEFV−	MEFV+	*P* value
				*n* = 5 (29.4)	*n* = 12 (70.6)	
Age (SD)			54.1 (12.1)	52 (13.6)	54.9 (11.9)	0.66
Male (%)			10 (58.8)	4 (80)	6 (50)	0.25
HLA (%)	A	A02	8 (47.1)	4 (80)	4 (33.3)	0.079
	B	B51	4 (23.5)	2 (40)	2 (16.7)	0.30
		B54	5 (29.4)	0 (0)	5 (41.7)	0.086
	C	Cw1	9 (52.9)	2 (40)	7 (58.3)	0.49
		Cw7	4 (23.5)	0 (0)	4 (33.3)	0.14
Symptoms (%)		Headache	14 (82.4)	2 (40)	12 (100)	0.003
		Oral mucosal lesions	13 (76.5)	3 (60)	10 (83.3)	0.30
		Cutaneous lesions	11 (64.7)	4 (80)	7 (58.3)	0.39
		Ocular lesions	1 (5.9)	1 (20)	0 (0)	0.11
		Gait disturbance	11 (64.7)	4 (80)	7 (58.3)	0.39
		Cognitive impairment	6 (35.3)	2 (40)	4 (33.3)	0.79
		Seizure	6 (35.3)	1 (20)	5 (41.7)	0.39
		Vascular manifestations	4 (23.5)	0 (0)	4 (33.3)	0.14
		Joint pain	11 (64.7)	3 (60)	8 (66.7)	0.79
		Exertional leg pain	8 (47.1)	0 (0)	8 (66.7)	0.012
MRI (%)	Sites	Brain stem	11 (64.7)	3 (60)	8 (66.7)	0.79
		Basal ganglia	4 (23.5)	2 (40)	2 (16.7)	0.30
		White matter	10 (58.8)	1 (20)	9 (75)	0.036
		Non‐parenchymal lesion	6 (35.3)	0 (0)	6 (50)	0.049

MEFV, Mediterranean fever gene; HLA, human leukocyte antigen; MRI, magnetic resonance imaging; SD, standard deviation.

## Discussion

Patients with CNS inflammation and symptoms related to NBD or NSD frequently exhibited *MEFV* gene mutations. Furthermore, patients with *MEFV* mutations commonly experienced headaches, exertional leg pain, and sustained white‐matter lesions without site predilection and non‐parenchymal lesions.

Single heterozygous mutations in the *MEFV* gene are common and were found to occur in one‐quarter of patients diagnosed with FMF.^5^ Our results showed that most (>70%) patients with NBD or NSD had *MEFV* gene mutations. This result supports the hypothesis that *MEFV* gene mutations may act as disease modifiers in NND. The most frequent mutations in Japanese patients with FMF included E148Q (40.2%), M694I (21.0%), L110P (18.8%), P369S (5.4%), and R408Q (5.4%).^12^ In our study, the most common *MEFV* gene mutations were E148Q (52.9%), R202Q (11.8%), P369S (11.8%), R408Q (11.8%), E84K (5.9%), G304R (5.9%), and L110P (5.9%). Headaches and exertional leg pain are often observed in patients with FMF.^5^, ^7^, ^13^ Some mutations reported in Japanese patients with FMF were also observed in patients in our study; therefore, we speculated that the mutations observed in our study contributed to the onset of FMF‐associated symptoms, including headaches and exertional leg pain.

Although serotypes of HLA class I and *MEFV* gene mutations showed no significant associations, B54 showed relatively high enrichment among patients with *MEFV* gene mutations (*P* < 0.10). The HLA class I B54 allele and *MEFV* gene mutations may additively contribute to onset of CNS inflammation.

The MRI lesions of patients who were *MEFV*+ disproportionately occurred in the white matter without site predilection. The typical predilection sites of NBD are brainstem and basal ganglia.^1^ Non‐parenchymal lesions such as cerebral venous thrombosis and acute meningeal syndrome have been reported as findings of non‐parenchymal NBD.^1^ White matter lesions without site predilection have been reported as one of the findings of NSD.^2^ Our study suggests that patients with CNS inflammation and white matter lesions without site predilection or non‐parenchymal lesions have a potential auto‐inflammatory background associated with *MEFV* gene mutations.

While HLA is mainly associated with adaptive immune responses, the inflammasome machinery is present in numerous cell types and contributes to innate immune activation in multiple organs and in the CNS.^14^ Inflammasomes are cytosolic protein complexes that sense specific infectious or host stimuli and initiate inflammatory responses through caspase activation. Neurons are known to express several inflammasome proteins, including the nucleotide oligomerization domain, leucine‐rich repeat, and pyrin domain‐containing protein (NLRP) 1, NLRP3, and are absent‐in‐melanoma 2. The NLRP3 inflammasome is the most abundant inflammasome in the CNS and is a key contributor to neuroinflammation across a broad spectrum of nervous system disorders.^14^ It has been hypothesized that the pyrin protein suppresses the activation of procaspase‐1 and therefore interferes with NLRP3 inflammasome activation.^15^ Considering that pyrin is encoded by the human *MEFV* gene,^16^ our results suggest that CNS inflammation and symptoms related to NBD and NSD might be associated with dysfunction of pyrin‐dependent regulation of the inflammasome. Although this is the first cohort study investigating *MEFV* gene mutations in NBD and NSD, *MEFV* gene mutations have also been reported in multiple sclerosis.^17^ Mutations of *MEFV* gene may be involved in CNS‐inflammation in various neurological diseases.

In our study, one patient diagnosed as probable NSD with *MEFV* mutation was remarkably responsive to colchicine. Colchicine is a treatment for FMF caused by *MEFV* gene mutations. In patients with CNS inflammation with *MEFV* mutations, colchicine may be an effective treatment option. Large cohort studies including other diseases manifesting neurological inflammation are warranted to clarify the relationship between *MEFV* gene mutations and neurological inflammatory diseases.

## Author Contributions

HI contributed to the conception and design of the study, acquisition, and analysis of data; drafted the initial draft of the report and revised and edited the manuscript. YI was involved in the design of the study, contributed to the interpretation of the findings, and revised and edited the manuscript. AN, YN, KM, NK, AM, KT, YH, HM, AOI, and AT were involved in the design of the study, acquisition of the data, and revision and editing of the manuscript. DK was involved in *MEFV* gene analysis and edited the manuscript. AS and HT were involved in the design of the study and contributed to the interpretation of the findings and critical revision of the manuscript.

## Conflict of Interest

The authors declare no conflicts of interest.

## References

[1] Kalra S , Silman A , Akman‐Demir G , et al. Diagnosis and management of Neuro‐Behcet's disease: international consensus recommendations. J Neurol 2014;261:1662–1676. 24366648 10.1007/s00415-013-7209-3 PMC4155170

[2] Hisanaga K , Iwasaki Y , Itoyama Y . Neuro‐Sweet disease: clinical manifestations and criteria for diagnosis. Neurology 2005;64:1756–1761. 15911805 10.1212/01.WNL.0000161848.34159.B5

[3] Hisanaga K . Neuro‐neutrophilic disease: neuro‐Behçet disease and neuro‐Sweet disease. Intern Med 2007;46:153–154. 17301507 10.2169/internalmedicine.46.0175

[4] Giza M , Koftori D , Chen L , Bowness P . Is Behcet's disease a 'class 1‐opathy'? The role of HLA‐B*51 in the pathogenesis of Behcet's disease. Clin Exp Immunol 2018;191:11–18. 28898393 10.1111/cei.13049 PMC5721257

[5] Alghamdi M . Familial Mediterranean fever, review of the literature. Clin Rheumatol 2017;36:1707–1713. 28624931 10.1007/s10067-017-3715-5

[6] Wu Z , Zhang S , Li J , et al. Association between *MEFV* mutations M694V and M680I and Behcet's disease: a meta‐analysis. PLoS ONE 2015;10:e0132704. 26176758 10.1371/journal.pone.0132704 PMC4503748

[7] Ishikawa H , Shindo A , Ii Y , et al. Vertebral artery dissection associated with familial Mediterranean fever and Behcet's disease. Ann Clin Transl Neurol 2019;6:974–978. 31139696 10.1002/acn3.773 PMC6529923

[8] Jo T , Horio K , Migita K . Sweet's syndrome in patients with MDS and MEFV mutations. N Engl J Med 2015;372:686–688. 10.1056/NEJMc1412998 25671271

[9] Ishikawa H , Shindo A , Ii Y , et al. Mediterranean fever gene mutations in patients with possible neuro‐Sweet disease: a case series. J Neurol Neurosurg Psychiatry 2018;89:1119–1121. 29175894 10.1136/jnnp-2017-316667

[10] Watad A , Tiosano S , Yahav D , et al. Behcet's disease and familial Mediterranean fever: two sides of the same coin or just an association? A cross‐sectional study. Eur J Intern Med 2017;39:75–78. 27776949 10.1016/j.ejim.2016.10.011

[11] International Study Group for Behcet's Disease . Criteria for diagnosis of Behcet's disease. Lancet 1990;335:1078–1080. 1970380

[12] Kishida D , Nakamura A , Yazaki M , et al. Genotype‐phenotype correlation in Japanese patients with familial Mediterranean fever: differences in genotype and clinical features between Japanese and Mediterranean populations. Arthritis Res Ther 2014;16:439. 25261100 10.1186/s13075-014-0439-7 PMC4201677

[13] Sugie M , Ouchi T , Kishida D , Yasaki S . Atypical type of familial Mediterranean fever: an underdiagnosed cause of chronic aseptic meningitis. Neurol Clinical Neurosci 2018;6:191–193. 10.1111/ncn3.12232 PMC6282518 30546872

[14] Mamik MK , Power C . Inflammasomes in neurological diseases: emerging pathogenic and therapeutic concepts. Brain 2017;140:2273–2285. 29050380 10.1093/brain/awx133

[15] Campbell L , Raheem I , Malemud CJ , Askari AD . The relationship between NALP3 and autoinflammatory syndromes. Int J Mol Sci 2016;17:E725. 27187378 10.3390/ijms17050725 PMC4881547

[16] Heilig R , Broz P . Function and mechanism of the pyrin inflammasome. Eur J Immunol 2018;48:230–238. 29148036 10.1002/eji.201746947

[17] Vidmar L , Maver A , Drulovic J , et al. Multiple Sclerosis patients carry an increased burden of exceedingly rare genetic variants in the inflammasome regulatory genes. Sci Rep 2019;1:9171. 10.1038/s41598-019-45598-x PMC6591387 31235738

